# The Domestic Staff—IV

**Published:** 1908-09-19

**Authors:** 


### Nursing Administration.

### THE DOMESTIC STAFF—IV.

### MEALS AND RECREATION.

It is a great mistake to suppose that servants dislike a strict mistress. On the contrary, the most contented households are those in which no breach of duty is overlooked, and wherein a wholesome back bone of something akin to fear exists in the staff with regard to their housekeeper. When they have once learned to recognise that her ideal of duty performed is a high one, and that no slacking is tolerated, they are not slow to find that this attitude of hers lends dignity to everyday routine. But the housekeeper cannot rest content with getting the machine to work well during duty hours. The members of her domestic staff are very human, responsive to influence, easily led astray, very lively and eager for pleasure, even if they can be made keen about their work, and with almost boundless capacity for improvement. What is she going to do with this troublesome, many-sided, interesting group of young women? Many of them come from indifferent homes and have the elements of gentle breeding all to learn. They have to be taught to modulate their voices, to regard horse-play as bad form, to be restrained in speech and to drop uncouth gestures and rough manners. When once the right tone has been set, the seniors perform the office of training in these matters for the newcomers as they appear. A higher social order is inaugurated, and since "we needs must love the highest when we see it," the desire to improve herself is a strong feature in all girls of a nice disposition, and can be counted on as a valuable factor in the situation. Should the wrong kind of girl get into the staff, a girl who continues to rebel against rule, she must be eliminated, but the number of failures will grow smaller as the tone improves, and often the least promising beginners turn into the best servants in the end.

Meals are, perhaps, amongst the strongest civilising forces we possess. If housekeepers would only take a little more trouble about the serving and supervision of the maids' meals, they would have less need to grumble about their behaviour at other times. In all institutions the meals should be conducted with the orderly serenity which prevails in the nurses' quarters, and to ensure this the housekeeper herself, or a sister in charge over the maids should preside. At Guy's Hospital, where the domestic staff in the nurses' home numbers over 90, the meals are presided over by the home sister, and a special feature is the spirited rendering of the musical grace. It is, of course, far easier to produce the right atmosphere when numbers contribute to formality. What generally happens is that the meals of the domestic staff are scrambled through rather in a rough-and-ready fashion, and that those among the number who would like to keep things on a higher level are a little at the mercy of those who do not care. This is rectified by the presence of some one in authority, whether it be a cook-housekeeper, or one of the sisters. Trouble bestowed in the direction of neat and orderly meals is well repaid and will be warmly appreciated by the maids who often fare worse than is at all suspected in institutions where their interests in this respect are left to chance.

The matron has not finished with off-duty hours when she has arranged the time-table and given each of her maids the opportunity of taking some outdoor exercise in the week during daylight hours. There is generally spare time in the evening among members of an institution staff which can be turned to profitable account if those in authority will lend a helping hand. In many institutions a sewing-machine is kept for the use of the maids, and dressmaking or millinery lessons in winter evenings are eagerly taken advantage of. Permission to attend continuation classes such as are available in most towns in the evening would often be gratefully received, and through this means promising girls whose education has been neglected can be encouraged to make up for wasted years. Anything which brings a tone of earnest purpose among girls whose daily duties are for the most part mechanical has a good effect, for that the domestic staff alone among all the purposeful workers in the institution should have no outlook is a condition of affairs which puts them out of touch with its pervading spirit. It is a waste of opportunity, and the institution suffers no less than the workers. It must never, however, be forgotten that young girls need an outlet for their superabundant spirits. They cannot be always at work, and if they ought to be always improving themselves half the time it must be under the disguise of amusement. The matron who dislikes the sight of a round back will institute a weekly course of physical exercises for her maids. Where the numbers are sufficient a choral class will be found very attractive, and a weekly dance among themselves serves to work off steam and promote good fellowship better than anything else. At Guy's Hospital the domestic staff are occasionally indulged with a fancy ball, expenditure on costumes being limited to 6½d. a head, with very pretty and often very instructive results.

Does all this sound very revolutionary? We believe it to be the natural consequence of the better education now available for the children of the artisan. Unless it can be proved to make for efficiency the administrator will look askance at such novelties as we have described. But when for confusion is substituted discipline, when incessant advertising for maids gives place to competition to enter a well-ordered service, when the domestic staff becomes a source of pride and contentment to the matron who has been wont to consider it her great scourge in life, then even the old-fashioned housekeeper may begin to think there is something in it. To our minds not the least valuable part of a domestic staff trained on good lines is the experience it gives to members of the training school in moulding raw material to serve a high purpose.

